# Effects of antibiotic cocktail on the fecal microbiota and their potential correlation of local immune response

**DOI:** 10.1186/s12866-024-03424-z

**Published:** 2024-07-31

**Authors:** Ting Liu, Yin Wang, Zhuoer Hou, Zhenyu Shi, Rongyun Wang, Yanan Shi, Lijiangshan Hua, Lingyun Wu, Min Xu, Xinghong Ding, Qiuhua Sun

**Affiliations:** 1 School of Basic Medical Sciences, Zhejiang Chinese Medical University, No.584, Binwen Road, Hangzhou, 310053 Zhejiang Province China; 2 School of Nursing, Zhejiang Chinese Medical University, Hangzhou, China; 3 The First Affiliated Hospital of Zhejiang Chinese Medical University (Zhejiang Provincial Hospital of Chinese Medicine), Hangzhou, China

**Keywords:** Antibiotic cocktail, Gut microbiota, Fecal microbiota, Local immune response, Mimicking germ-free mice

## Abstract

**Background:**

The guts of mammals are home to trillions of microbes, forming a complex and dynamic ecosystem. Gut microbiota is an important biological barrier for maintaining immune homeostasis. Recently, the use of antibiotics to clear gut microbiota has gained popularity as a low cost and easy-to-use alternative to germ-free animals. However, the effect of the duration of the antibiotic cocktail on the gut microbiome is unclear, and more importantly, the effect of dramatic changes in the gut microbiota on intestinal tissue morphology and local immune response is rarely reported.

**Results:**

We observed a significant reduction in fecal microbiota species and abundance after 1 week of exposure to an antibiotic cocktail, gavage twice daily by intragastric administration. In terms of composition, *Bacteroidetes* and *Firmicutes* were replaced by *Proteobacteria*. Extending antibiotic exposure to 2–3 weeks did not significantly improve the overall efficiency of microbiotal consumption. No significant histomorphological changes were observed in the first 2 weeks of antibiotic cocktail exposure, but the expression of inflammatory mediators in intestinal tissue was increased after 3 weeks of antibiotic cocktail exposure. Mendelian randomization analysis showed that *Actinobacteria* had a significant causal association with the increase of IL-1β (OR = 1.65, 95% CI = 1.23 to 2.21, *P* = 0.007) and TNF-α (OR = 1.81, 95% CI = 1.26 to 2.61, *P* = 0.001).

**Conclusions:**

Our data suggest that treatment with an antibiotic cocktail lasting 1 week is sufficient to induce a significant reduction in gut microbes. 3 weeks of antibiotic exposure can lead to the colonization of persistant microbiota and cause changes in intestinal tissue and local immune responses,

**Supplementary Information:**

The online version contains supplementary material available at 10.1186/s12866-024-03424-z.

## Background

Methods for obtaining gut microbiota-cleared mice include germ-free (GF) mice and antibiotic induced mock germ-free mice. GF mice are required to be significantly free of microorganisms, including microbiota, viruses, fungi, protozoa, and parasites, throughout their lifetime and are considered the gold standard for studying complete absence of microorganisms or for performing fecal microbiota transplantation experiments [1]. However, many researchers still do not have access to this model due to the need for specialized facilities and the high cost. The use of broad-spectrum antibiotic cocktail to deplete mouse gut microbiota to mimic a germ-free state is a low-cost alternative to GF mice [2].

Recent studies have described mouse models constructed with antibiotic exposure in which gut microbial communities were dramatically altered by antibiotic administration [3]. There is no doubt that, individual antibiotics are sufficient to perturb the ecological balance of gut microbes. For example, a single dose of clindamycin has been shown to cause chronic susceptibility to *Clostridium difficile* infection in mice [4]. Furthermore, pretreating mice with oral streptomycin not only significantly reduced the normal microbiota, but also led to an inflammatory intestinal response after oral infection with *Salmonella* [5]. However, multiple antibiotic mixtures were more effective at depleting gut microbiota. In particular, a combination regimen of antibiotics that targets a wide range of gut microbes, including both aerobic and anaerobic bacteria, is necessary for the construction of simulated GF mouse models [1]. Several studies have reported different antibiotic combinations and treatment regimens [3, 6]. Tirelle et al. [3]. compared different antibiotic regimens to provide a reference for the most suitable antibiotic cocktail for our research work to observe the multiple effects of this antibiotic cocktail on the intestinal ecology of mice.

The effect of antibiotics on the gut microbiota is well established, but it is difficult to fully predict, especially since changes in the abundance of gut microbes and the emergence of resistant microbiota or fungi are occur almost simultaneously on a temporal scale [7]. Although the study suggested that the fecal microbiota of mice was robustly depleted after four days of treatment with the antibiotic cocktail. even after the GF mice were modeled, the antibiotic cocktail still needed to be used sustainably to complete other interventions. The duration of the antibiotic exposure varies from 4 to 14 days [3, 6]. So does a longer antibiotics cocktail exposure lead to a higher microbial clearance? However, there is little information on the effects of longer antibiotic cocktail exposure on gut or fecal microbiota. In addition, a rising number of studies focusing on antibiotic exposure have shown that gut microbiota dysbiosis increases the risk for various diseases, such as antibiotic-associated colitis, inflammatory bowel disease, and celiac disease [8–10]. Importantly, more evidence is needed to understand the effects of the antibiotic cocktail, including the dual effects on gut microbiota and gut tissues, as well as their potential correlation of local immune response. This will provide important parameters for the establishment of GF mouse models and also provide insights into the effects of antibiotic exposure on intestinal tissues.

In this study, we used the classic antibiotic cocktail formulation (a mixture of ampicillin, neomycin, metronidazole, and vancomycin), administered orally twice daily. Gut microbiota changes and the abundance of specific microbiota taxa in the feces of antibiotic-treated mice were analyzed mainly by 16 S rRNA gene sequencing. We aimed to: (i) study changes in intestinal microbiota induced by antibiotic cocktail at different times (1 to 3 weeks), (ii) to detect changes in intestinal histomorphology and inflammatory response, and (iii) to identify specific changes in microbiota after antibiotic cocktail exposure at different times. (iv) to identify potential interactions between specific microbiota and host immunity (key inflammatory mediators). The results obtained will provide more comprehensive reference information for the construction of GF mice and provide important basis for the role of the microbiota in human disease.

## Results

### Effect of antibiotic cocktail on the richness and diversity of microbiota

**Fig. 1:**
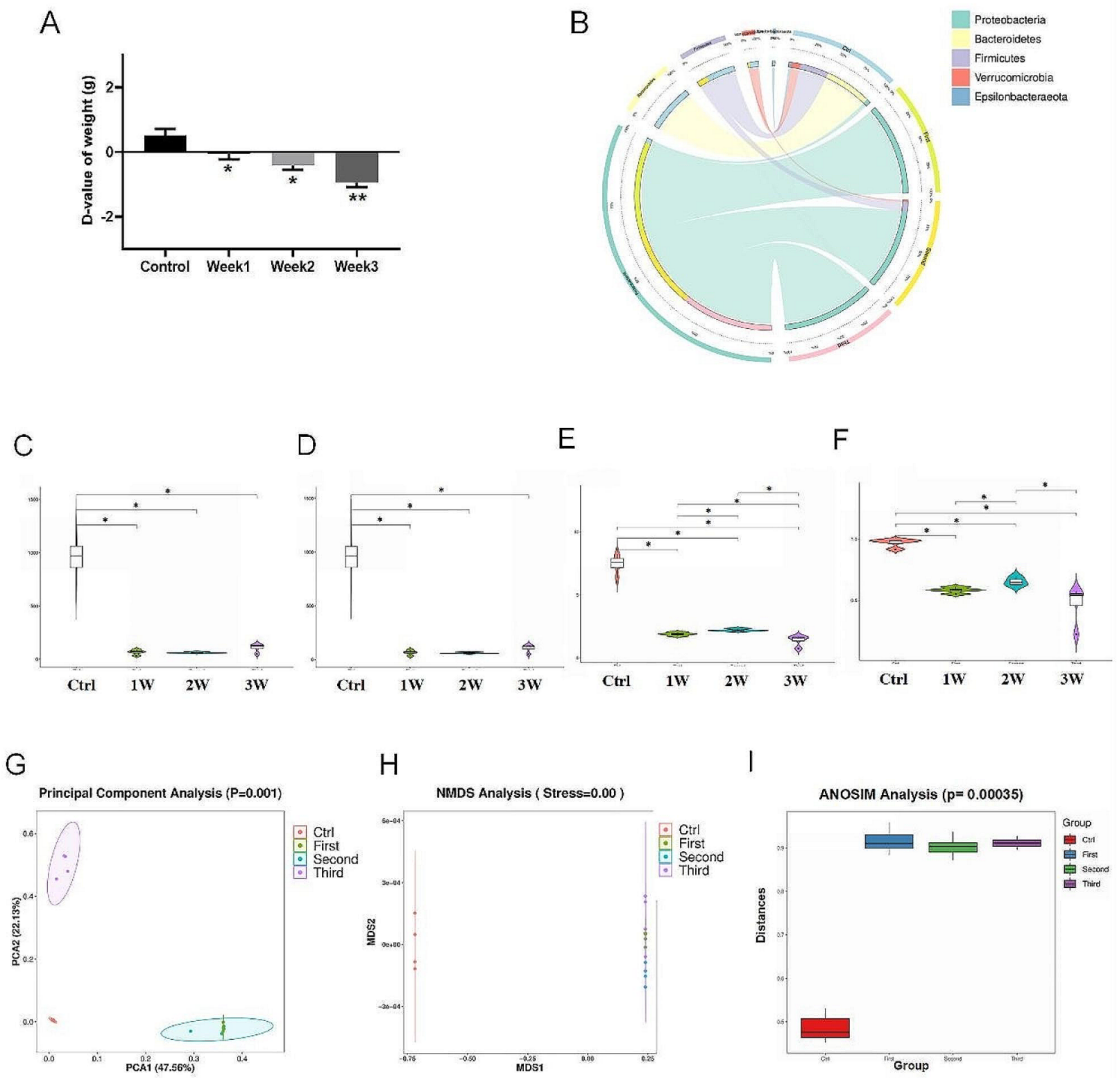
Effects of antibiotic cocktail on growth performance and microbiota. (**A**) D-value of body weight. D-value refers to final body weight minus initial body weight. (**B**) Circos plots of microbiome at phylum level. (**C**) Chao 1. (**D**) Observed OTUs. (**E**) Shannon. (**F**) Simpson. (**G**) Weighted unifrac PCA. (**H**) Weighted unifrac NMDS analysis (stress < 0.1). (**I**) ANOSIM distances to Control. *P < 0.5; **P < 0.01

After treating the mice with an antibiotic cocktail, there were significant changes in their growth performance and gut microbiome, regardless of the intervention for 1, 2, or 3 weeks. Weight loss in the mice exposed to the antibiotic cocktail was positively correlated with exposure time, as shown by the body weight D-value, compared to the normally grown mice. (Fig. 1A). At the same time, changes in stool morphology of mice after three weeks of antibiotic exposure were also observed. Due to the shape of the particles, the stools became loose and watery, exhibiting mild diarrhea symptoms. Further, we found that fecal specimens collected during antibiotic cocktail exposure showed severe depletion of the microbiota, characterized by a significant reduction in richness and diversity. Circos maps indicate microbial diversity at the generic level is impaired after 1 to 3 weeks of antibiotic cocktail exposure (Fig. 1B). We measured gut microbial alpha diversity using a generalized linear model through different methodologies. Chao1 and Observed OTUs reflect a significant reduction in the diversity of gut microbiota composition and distribution in mice induced by antibiotic cocktail therapy (*p* < 0.01) (Fig. 1C-D). Consistently, The Shannon index and Simpson index demonstrated similar tendencies and showed significant differences between the four groups (Fig. 1E-F) (*p* < 0.05). To extend our understanding of the role of microbiome diversity, we performed beta-diversity analysis to generate PCA and NMDS plots (Fig. 1G-H). Apparent clustering separation in the principal component revealed the different community structures between the four groups, suggesting that these communities are distinct in terms of their compositional structure. In addition, the Bray-Curtis metric distance increased with the duration of antibiotic exposure (Fig. 1I), indicating that the mice treated with antibiotics for 1 to 2 weeks were highly similar in the principal components of their gut microbiota. Conversely, the microbiota composition of mice treated for 3 weeks was already significantly different from either of the other two groups (Fig. 1G).

### Microbial alterations after the exposure of antibiotic cocktail

**Fig. 2:**
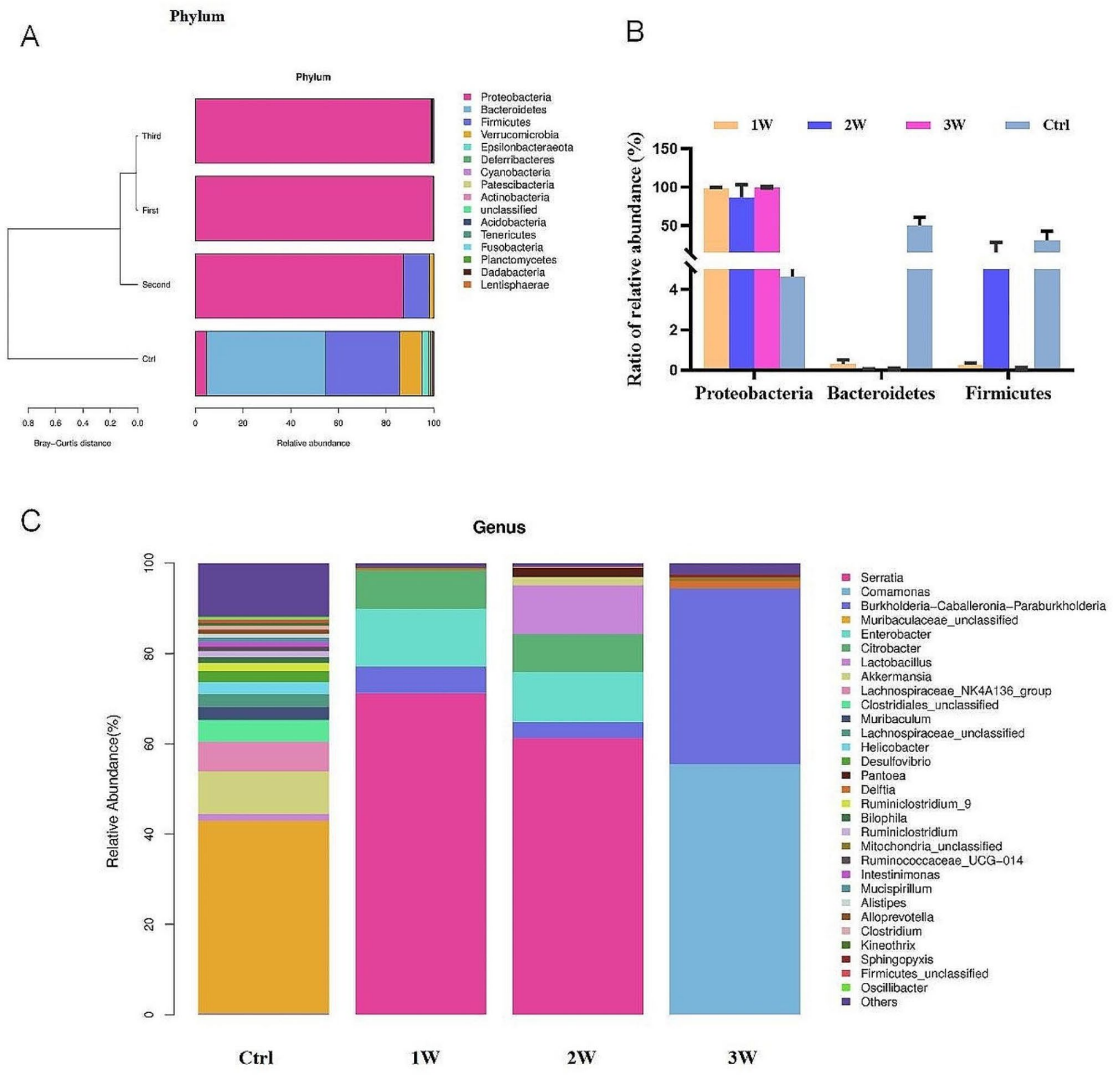
The relative abundance of fecal microbiota. (**A**) The relative abundance and Bray-Curtis distance of microbes across different groups. (**B**) The ratio of *Proteobacteria*,* Bacteroidetes* and *Firmicutes* in each group. (**C**) The relative abundance of fecal microbiota at the phylum level. The data are represented as mean ± SD

The microbial alterations were further analyzed. At the phylum level, *Bacteroidetes* (50.06%) and *Firmicutes* (30.99%) were the dominant bacteria in the gut microbiota of control mice. *Proteobacteria* showed absolute dominance after antibiotic exposure, reaching 99.74%, 87.18% and 98.83% of relative abundance in the 1-week, 2-week, and 3-week groups respectively. Interestingly, *Verrucomicrobia* (1.82%) occupied a certain abundance in the second week group, which was not observed in the other two groups (Fig. 2A and B). The taxonomic compositions between the control group and antibiotic-treated groups were also compared at the class/order/family level (Supplementary Figures S1-S3). At the genus level, following 1 to 2 weeks of antibiotic cocktail exposure, the proportion of *Serratia* (80.63%) was increased significantly and became the dominant genus. In the 3-week antibiotic-treated mice, the ratios of *Comamonas* (55.42%) and *Burkholderia* (38.89%) were the highest (Fig. 2C).

### Changes of intestinal histomorphology in mice after antibiotic cocktail exposure

**Fig. 3:**
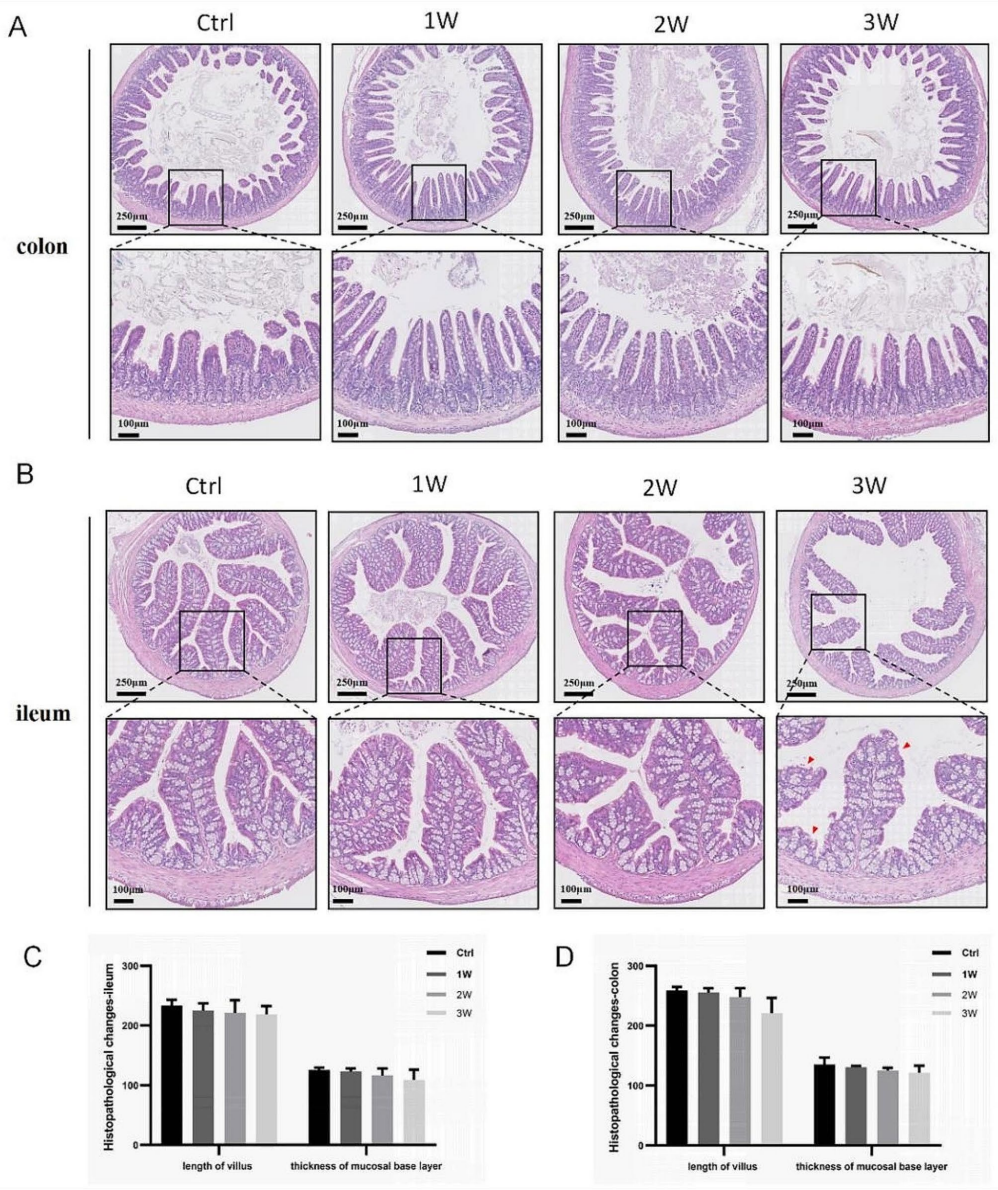
Histomorphologic changes of ileum and colon. (**A**) Representative images of colon with H&E staining. (**B**) Representative images of ileum with H&E staining. (**C**) Quantitative analysis of length of villus and thickness of mucosal basement layer for colon (*n* = 4). (**D**) Quantitative analysis of length of villus and thickness of mucosal basement layer for ileum (*n* = 4). Scale bar = 100–250 μm. The data are presented as means ± SD. *P < 0.5

In order to investigate the effects of gut microbiome alterations on intestinal issue morphology, H&E staining was performed on the ileum and colon sections of mice (Fig. 3). Histopathological examination revealed no significant changes in the ileum following 1 to 3 weeks of antibiotic exposure (Fig. 3A, C). However, after 3 weeks of exposure to the antibiotic cocktail, the crypt orifice of the colon was observed to be enlarged, and local columnar epithelial integrity was compromised. Nevertheless, no statistically significant differences were noted in the villus length and thickness of the mucosal base layer (Fig. 3B, D).

### Inflammation mediators in mouse colon after the exposure of antibiotic cocktail

**Fig. 4:**
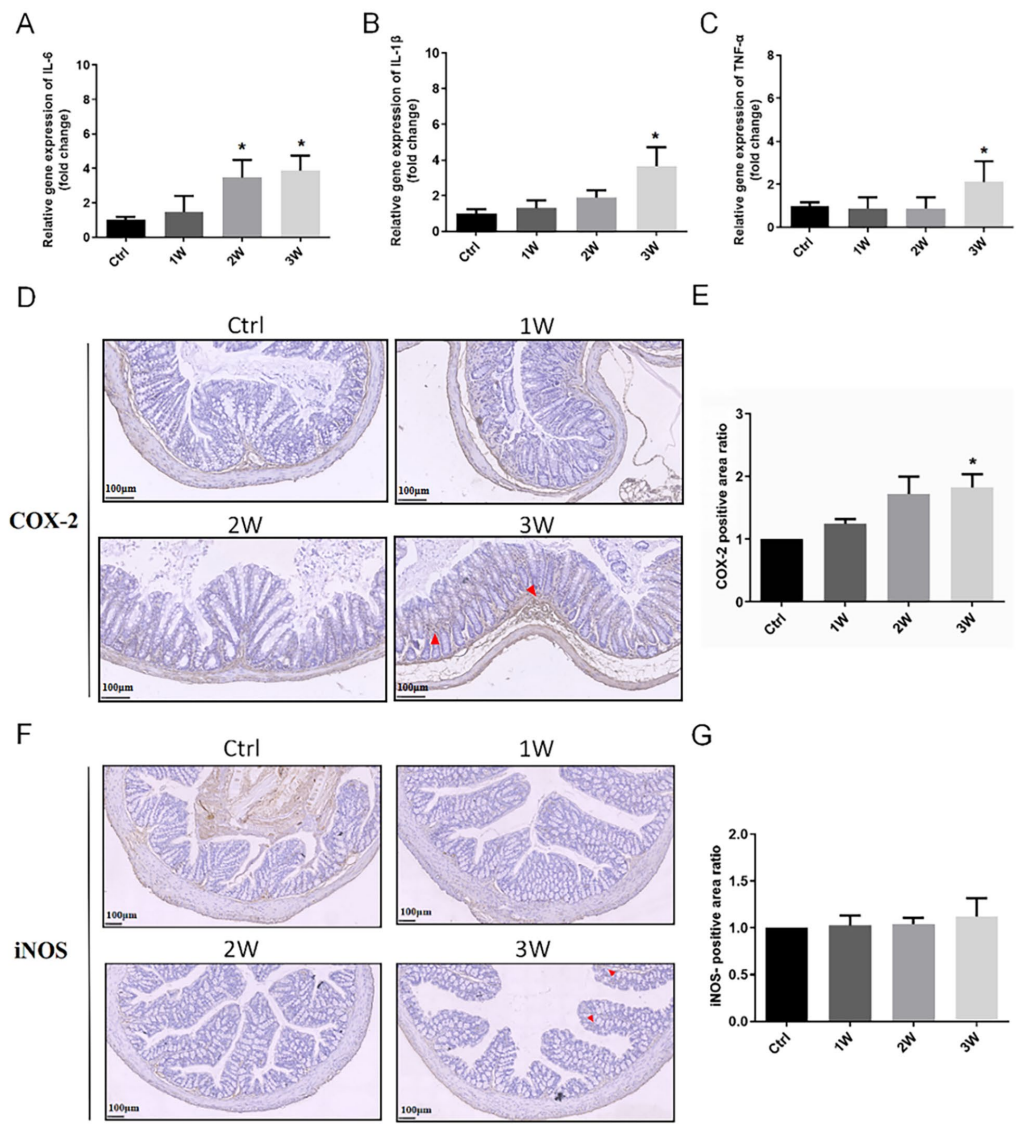
Changes of mRNA expression and protein activation of inflammatory mediators in colon. (**A**) Relative gene expression of IL-6 in colon. (**B**) Relative gene expression of IL-1β in colon. (**C**) Relative gene expression of TNF-α in colon. (**D**) Representative images of the expression of COX-2 in colon. (**E**) Quantitative analysis of COX-2 positive area (*n* = 4). (**F**) Representative images of the expression of iNOS in colon. (**G**) Quantitative analysis of iNOS positive area (*n* = 4). Scale bar = 100 μm. The data represented as mean ± SD. *P < 0.5; **P < 0.01

To investigate the impact of gut microbiota on the local immune response, we analyzed inflammatory mediator expression in colon tissues of mice from each group (Fig. 4). Results revealed elevated mRNA levels of IL-6, TNF-α and IL-1β in colon tissue following three weeks of antibiotic cocktail exposure, particularly for IL-6 (all *P* < 0.05) (Fig. 4A-C). In addition, no significant changes were observed in IL-4 and IL-12 (Supplemental Figure S4). Accordingly, the expression of pro-inflammatory protein COX-2 and the positive area ratio were augmented in colon tissues after 3 weeks of antibiotic cocktail exposure (*P* < 0.05) (Fig. 4D-E). However, iNOS expression remained unaltered in the antibiotic cocktail-exposed group, despite a slight increase in the third week, which was not statistically significant (Fig. 4F-G).

### The microbiota responsible for the alterations and their potential correlation

**Fig. 5:**
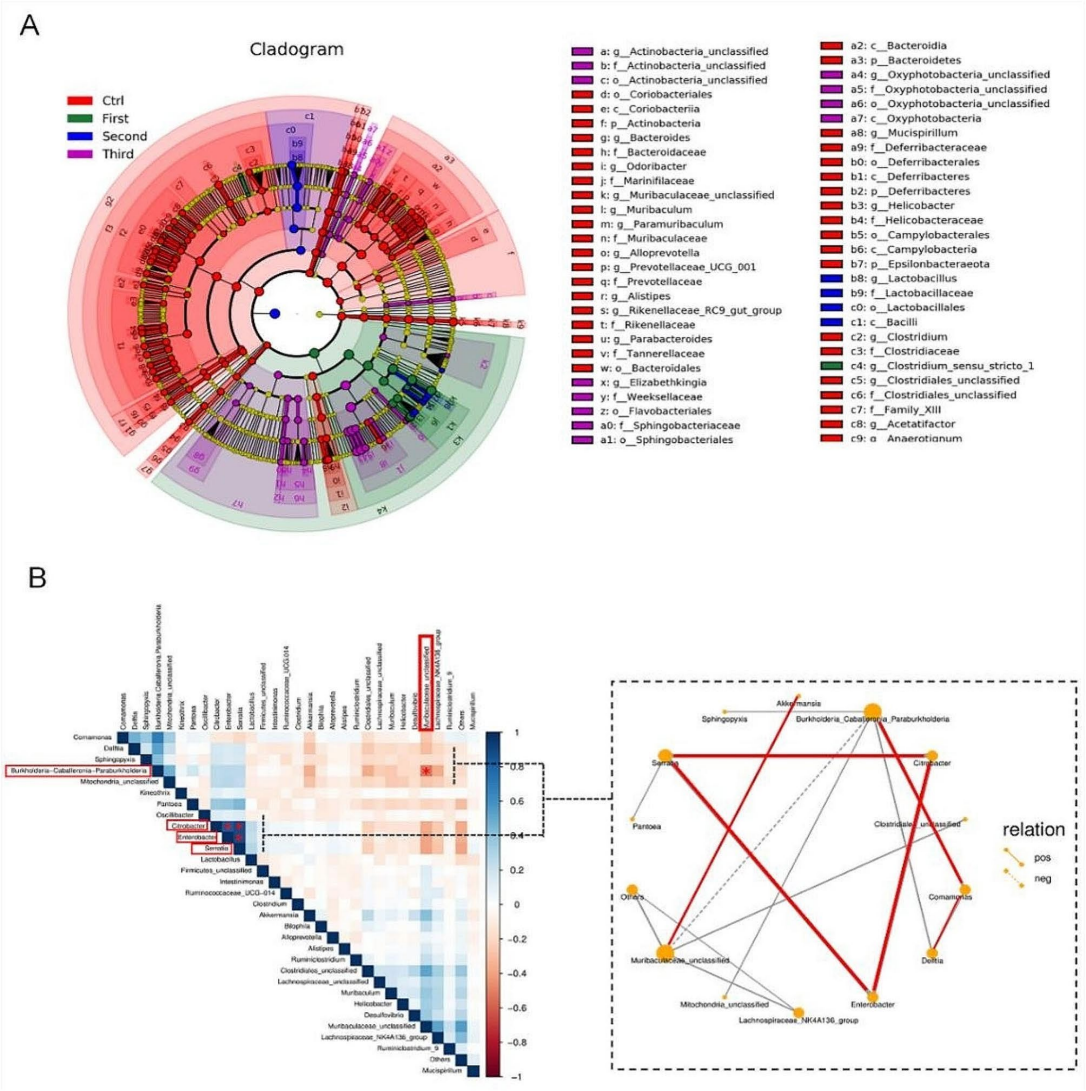
The nuclear microbiota is responsible for differences in the composition of microbiota. (**A**) A taxonomic cladogram obtained using LEfSe. The red, green, blue, and purple nodes represent the microbiota that played a significant role in the Ctrl group, the 1-week group, the 2-week group and the 3-week group, respectively. (**B**) Species Correlation Network. The heat map visualizes the correlation through color blocks. The nodes of the network graph represent different dominant genera, and thicker lines indicate a stronger correlation; solid lines represent positive correlation, while dashed lines represent negative correlations. By default, only relationships with a correlation coefficient RHO > 0.4 are displayed (*P* < 0.05)

To further elucidate the microbiota responsible for alterations in microbiota composition and inflammation mediators in the colon after antibiotic exposure, LEfSe and biological relationship heatmaps were used to analyze multi-level species differences. According to the analysis between the two groups, the genus *Lactobacillus* (spanning from the class *Bacilli* to the family *Lactobacillaceae*) emerged as the primary microbiota contributing to gut microbiota dysbiosis in the 2-week antibiotic-treated group. Conversely, *Actinobacteria* and *Oxyphotobacteria* accounted for the the compositional differences in the microbiota of 3-week antibiotic-treated mice (Fig. 5A). Furthermore, intricate dependencies exist among gut microbiota. Specifically, *Serratia*,* Enterobacter*, and *Citrobacter* form a positive regulatory loop, as well as *Burkholderia*,* Comamonas*, and *Delftia* exhibit a positive correlation. In contrast, *Burkholderia* and *Muribaculaceae* were negatively regulated (Fig. 5B).

### Causal effects of differential microbiota on intestinal inflammatory mediators

**Fig. 6:**
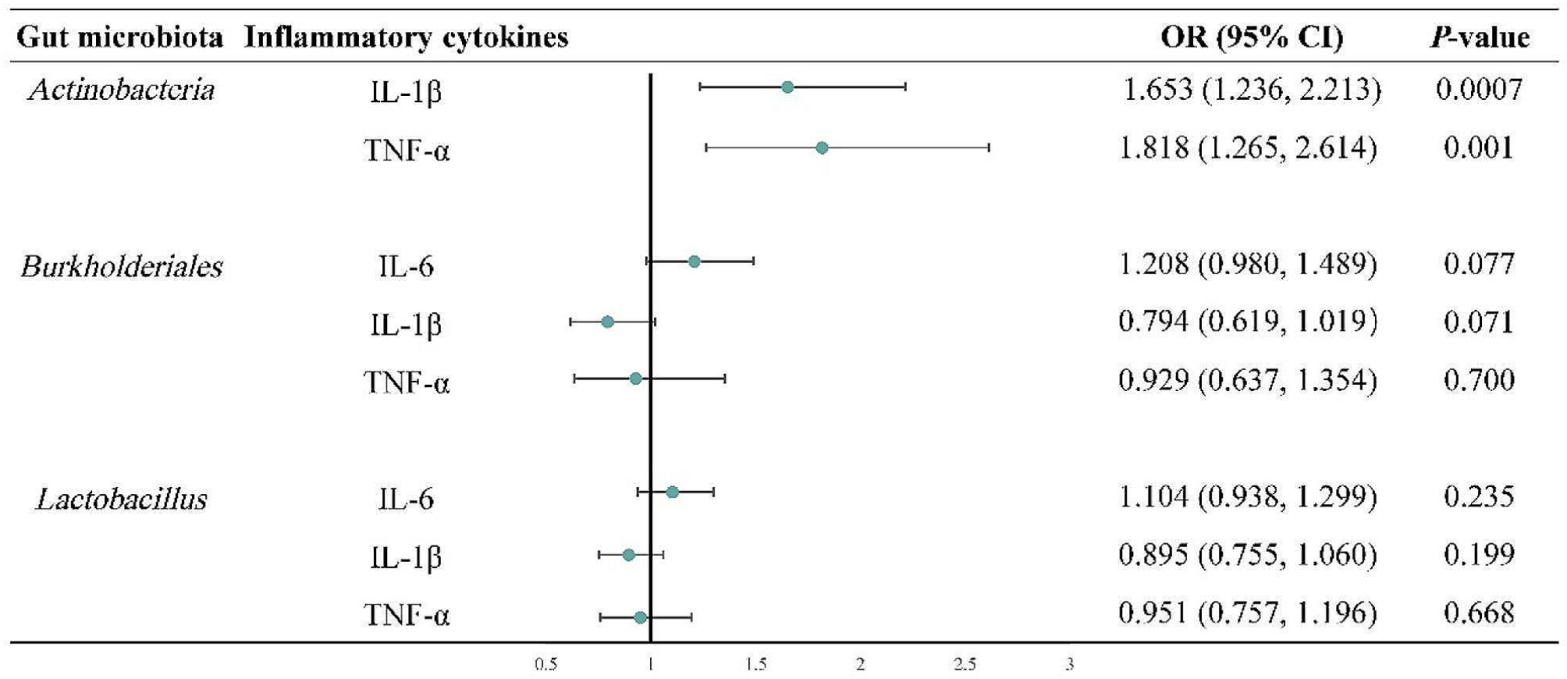
Associations between genetically predicted gut microbes and IL-6, IL-1β, and TNF-α. CI: confidence interval; IL: interleukin; TNF-α: Tumor necrosis factor-α

To further analyze the potential impact of differential microbiota on intestinal inflammatory mediators, we used Mendelian Randomization (MR) for further examine. We identified SNPs related to *Actinobacteria* with a significance threshold of *P <* 5e-6. These SNPs are detailed in the supplementary materials (Supplementary Table S1). Except for the first SNP (rs10752777), the others were strongly associated with IL-6 (refer to website: http://www.phenoscanner.medschl.cam.ac.uk/). This indicates a significant overlap of SNPs in *Actinobacteria* and IL-6. Therefore, an increase in *Actinobacteria* levels can lead to an increase in IL-6 levels. Additionally, inverse variance-weighted (IVW) results suggest that genetically predicted *Actinobacteria* have a causal effect on the increase of IL-1β (OR = 1.65, 95% CI = 1.23 to 2.21, *P* = 0.007, Fig. 6) and TNF-α (OR = 1.81, 95% CI = 1.26 to 2.61, *P* = 0.001, Fig. 6). Simultaneously, the weighted median approach results also support the causal effect of *Actinobacteria* on higher levels of IL-1β (Supplementary Table S2). However, IVW did not find a causal association between *Burkholderia* or *Lactobacillus* on changes in levels of IL-6, IL-1β, and TNF-α (*P* > 0.05, Fig. 6).

MR Analysis showed that horizontal pleiotropy and heterogeneity were absent (Supplementary Tables S3 and S4), and the leave-one analysis showed that no single SNP that drove or contributed to the bias in the results (Supplementary Figure S5-S8). Detection using the “MR-PRESSO outlier test” revealed no abnormal outlier SNPs. After excluding SNPs with potential confounding variables (Supplementary Table S5), the results remained unchanged (Supplementary Table S2), further verifying the stability of the findings in this study.

## Discussion

In this study we adopted a classic scheme that broadly targets Gram-positive, aerobic, and anaerobic bacteria. The formula includes vancomycin (a glycopeptide antibiotic), ampicillin (a β-lactam antibiotic), neomycin (an aminoglycoside antibiotic) and metronidazole (a nitroimidazole antibiotic) [3]. Intraperitoneal injection, oral administration, and gavage are the most common modes of administration. Intraperitoneal administration had a pronounced acute effect, but oral administration had a longer effect than injection [6, 11]. Oral antibiotics mixed with water is a relatively convenient route of administration. However, the mixing of multiple antibiotics leads to a decrease in the taste of drinking water, and the water consumption of the treated mice is reduced [6]. Therefore, in this study, the antibiotic cocktail was administered intragastrically to ensure that the mice received the same amount of antibiotic exposure.

antibiotic exposure in mice reproduced the effects and long-term changes in the human gut microbiota, including dramatic declines in diversity and representation of specific taxa and increases in antibiotic-resistant strains [10, 12]. *Firmicutes*, *Bacteroidetes* and *Verrucomicrobia* were largely replaced by *Proteobacteria* in mouse feces after exposure with the antibiotic cocktail. This is consistent with observations from similar studies [3, 7, 13]. Interestingly, *Firmicutes* species still maintained a proportion of 10.91% in the second week of antibiotic exposure, while almost all were replaced by *Proteobacteria* after the third week of exposure. Further observations at the genus level combined with the relationship diagram may explain this phenomenon. *Serratia* (71.19%), *Enterobacter* (12.80%), *Burkholderia* (5.82%) and *Citrobacter* (8.59%) became the dominant genera after antibiotic shock exposure (1st week). The positive regulatory relationship between *Serratia*, *Enterobacter* and *Citrobacter* likely contributed to this trend. Additionally, the negative regulation of *Muribaculaceae* by *Burkholderia* may have further influenced it. When the antibiotic cocktail continued to the second week, these genera continued to be depleted, and their scores decreased, and the *Lactobacillus* belonging to the *Firmicutes* phylum increased from 0.01 to 10.87%. Notably, *Muribaculaceae* and members, as major mucin monosaccharide foragers, have been reported to have the potential to prevent *C. difficile* from acquiring mucosal sugars and thereby reduce pathogen colonization [14]. The antibiotic cocktail continued until the third week, and *Comamonas* (55.42%) and *Burkholderia* (38.89%) belonging to *Proteomicrobiota* became the most dominant microorganisms. *Comamonas* and *Burkholderia* and their strains are frequently reported to have intrinsic multidrug resistance (MDR), and they are common opportunistic pathogens [15, 16]. In addition, *Delftia*, belonging to the family of *Comamonasaceae*, exhibited a relative abundance of 1.63% by the third week. Recent studies have reported *Delftia* as a potential diagnostic marker in patients with drug-resistant tuberculosis [17].

Diet and feeding environment are also key conditions affecting the composition of gut microbes. Bidot et al. [18]. found that different combinations of bedding and water purification methods led to different gut microbiota, especially, samples from mice that received autoclaved water without additional treatment had the lowest gut microbiota richness. Ericsson et al. [19]. found that the interaction between different housing conditions and bedding materials had a significant effect on gut microbiota of mice, with the jejunal and cecal contents being the most affected. All mice in this study lived in individually ventilated cages that provided drinking water, and the animals’ diet contained crude protein (200 g/Kg), crude fat (60 g/Kg), crude fiber (50 g/Kg), phosphorus (6–12 g/Kg), calcium (10–18 g/Kg), lysine (13.2 g/Kg), methionine and cystine (7.8 g/Kg). The feeding conditions in this study were similar to those reported by Tirelle et al. [3]. We speculate that differences in the initial gut microbiota of mice exposed to a high-dose cocktail of antibiotics may not be sufficient to alter the dramatic effects of antibiotics on the microbiota. Moreover, since these mice were raised in the same environment, we would normally expect the effect to be uniform. Nevertheless, we still need to acknowledge that the effects of feeding environment and diet on the gut microbiota of animals are unexpected. In particular, transgenic mice or nude mice need to pay more attention to the effects of external variables (bedding, cage ventilation, and diet) on the intestinal microbiome composition of experimental mice.

The results of this study indicate that longer than 2 weeks use of antibiotic mixtures not only causes dysregulation of the microbiome, but also increases the expression of IL-6, IL-1β, TNF-α, and COX-2 in intestinal tissue. At the same time, we observed that intestinal tissue changes correlated with antibiotic exposure time. We suspect that this effect may be related to specific persistent microbiota. Further MR Studies further revealed that *Actinobacteria* were the key microbiota that led to increased expression of inflammatory mediators, although the correlation between IL-6 and COX-2 could not be shown on the forest plot. However, when we are trying to design treatments to kill gut microbes, it is important to consider the nature of antibiotic persisters. Combining the characteristics of Shultis et al. [20] for the definition of persistent and tolerant bacteria, they can be defined as persistent bacteria in this study, as they exist alone and form part of a small subpopulation. It has been suggested that some microbiota can adapt to changes in their environment, including entering a state of metabolic inactivity that renders antibiotic combinations ineffective or weak [21]. This may explain why *Actinobacteria* become resistant in the third week. Nevertheless, we don’t yet know how long after antibiotic exposure the mice’s gut microbes can adapt to their environment. Moreover, the list of tolerant microbiota may also change with longer antibiotic exposure, and future studies will provide more information.

The destruction of intestinal local immune homeostasis is not only caused by some specific drug-resistant microbiota, but antibiotics are also likely to contribute to this process. Several studies have suggested that antibiotic exposure is a major risk factor for the development of diarrhea and colitis [4, 5, 8, 22]. In addition to the direct drug toxicity of antibiotics, the depletion of endogenous microbiota and subsequent pathogen overgrowth are also major causes of diseases [10]. For example, *Bacteroidaceae* and *Lactobacillus* are the key microbiota types leading to DSS-induced intestinal flora imbalance in colitis, and *Clostridium difficile* and *Klebsiella oxytoca* are likely to be the key causes of colitis [4, 9]. This seems to explain the altered fecal morphology and weight loss observed in mice with prolonged antibiotic exposure in the present study. In addition, a balanced interaction between intestinal flora and the mucosal immune system is necessary for the maintenance of gastrointestinal homeostasis [9, 13, 23]. For example, gut microbiota has a profound effect on the balance of effector helper T cells (Th) and regulatory T cells (Treg) [9]. Similarly, Suzana et al. [24]. have shown that oral antibiotic combination treatment early in the life of dark agouti rats can disturb the intestinal microbiota, and the changes in the intestinal microbiota can lead to the escalation of central nervous system-directed autoimmunity and enhanced inflammatory expression in dark agouti rats, which is mediated by the disturbance of Th/Treg balance. It is likely that the antibiotic cocktail perturbs the balanced interactions between the native microbiota and the host and explaining the increased inflammatory expression in intestinal tissues and changes in intestinal homeostasis induced by antibiotic administration observed in clinical and experimental settings. In summary, there may be multiple potential reasons for the effects of antibiotics on intestinal tissue morphology and local immune response, such as the direct drug toxicity of antibiotics, the depletion of endogenous microorganisms, secondary over-proliferation of pathogenic microbiota, and the disruption of the balance between microorganisms and intestinal mucosal immunity [8, 10, 24]. However, to date our understanding of the effects and exact nature of the changes that cause microbial structure alteration is still very limited, and this will be the direction of our future research efforts and exploration.

### Limitation

There are some shortcomings in this study. First, due to the limitations of research resources and animal welfare, we only set up 16 mice in our study. Although small intra-group differences did not affect the results of this study, in view of the individual differences and statistical representativeness of animals, it is extremely necessary to increase the number of animal samples. Future studies may consider setting a more adequate sample size to reduce the impact of individual differences on on the results. Second, the study did not adequately compare the effects of different modes of administration (oral drinking water or gavage) and different feeding conditions (bedding, water, feed, and cage ventilation) on host gut microbes, and these are all factors that are known to affect the gut microbiome. Third, we only observed the effects of the antibiotic cocktail on gut microbes at third week, which is a relatively long-term (three-week) concept for our study. However, we believe that with prolonged antibiotic exposure, new discoveries may be made about the effects of antibiotic cocktail on gut microbiota.

## Conclusions

In conclusion, this study delves into the effects of antibiotic exposure on gut microbiota and intestinal local immune responses in mice, employing classic antibiotic cocktail formulations. Our findings indicate that a 1-week exposure to the antibiotic cocktail profoundly diminished microbial diversity. Furthermore, a 3-week antibiotic exposure facilitated the specific colonization of gut microbiota with MDR properties, encompassing *Serratia*, *Enterobacteriaceae*, *Burkholderia*, and *Common Amonas*. Additionally, this prolonged exposure exerted an impact on intestinal tissue, potentially stemming from the disruption of the immune balance between gut microbiota and the intestinal mucosa, ultimately leading to heightened expression of inflammatory mediators. Notably, *Actinobacteria* were the primary culprit behind the elevated expression of IL-1β and TNF-α. However, our study may not fully elucidate the intricate mechanism underlying the altered intestinal immune response triggered by the antibiotic cocktail.

## Methods

### Animals

C57BL/6 male mice (6–8 weeks old, 20–22 g) were purchased from Shanghai BK Experimental Animal Co., Ltd (Shanghai, China). All animals were raised in specific pathogen-free (SPF) conditions of the Laboratory Animal Center of our university. Feeding environment details: Room temperature was maintained at 22–24℃, relative humidity at 40–60%, and a 12 h light/dark cycle was observed. Each of the four mice was housed in a separate room with individually ventilated caging. Euthanasia of the animals was performed by intraperitoneal injection of pentobarbital sodium at a dose three times higher than the anesthetic dose. The loss of consciousness was rapid, followed by cessation of respiration and heartbeat, confirming euthanasia. All procedures were carried out in accordance with the guidelines of the NIH Guide for the Care and Use of Laboratory Animals, as well as the regulations of the American Veterinary Medical Association (AVMA) regarding euthanasia, and they were approved by the Committee of Zhejiang Chinese Medical University (Approval No. IACUC-20220221-11).

### Experimental design

**Fig. 7:**
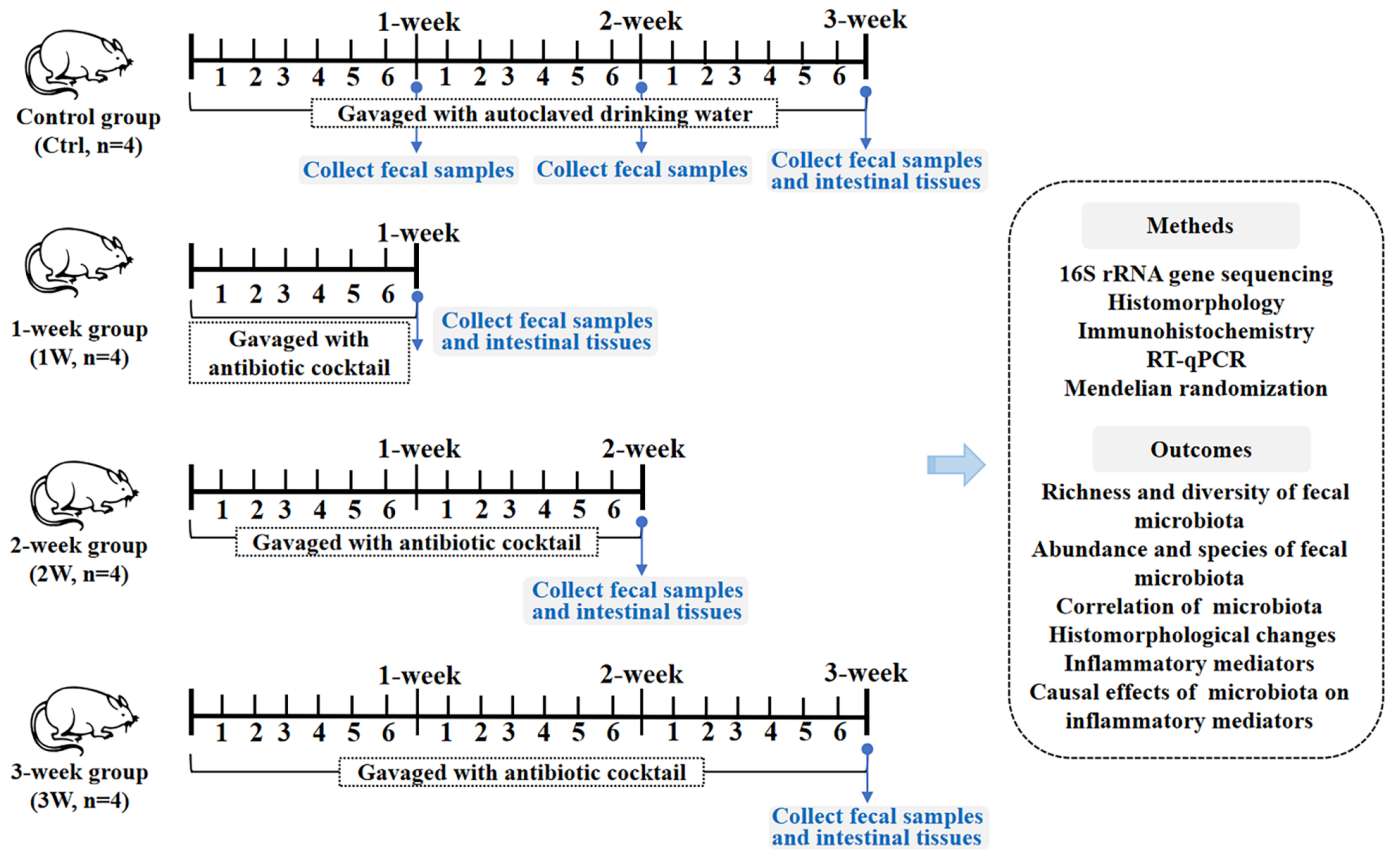
The flow chart of the experimental procedure and timeline

A total of 16 mice were randomly assigned to control (Ctrl) group or treatment groups (1- to 3-week). All the mice were raised in rooms with corn cob bedding (Domi Agricultural Technology Co., Ltd, China) and provided with drinking water and feed for laboratory mice (SPF grade, Medicine, Ltd, China). They were fully allowed to move around, eat, and drink water. Mice in the treatment groups were given 10 µL/g of the antibiotic cocktail by gavage twice daily with an 8-hour interval for 1 week (1 W), 2 weeks (2 W) and 3 weeks (3 W), respectively. Mice in the control group were gavaged with autoclaved drinking water for 3 weeks with the same intervention frequency. The combination scheme and administration method of the antibiotic cocktail refers to the research of Pauline Tirelle et al. [3]. According to their report, the antibiotic cocktail contained 10 mg/mL Ampicillin (Sigma, St. Louis),10 mg/mL Neomycin trisulfate salt hydrate (Aladdin, China), 10 mg/mL Metronidazole (Sigma, St. Louis) and 5 mg/mL Vancomycin hydrochloride (Sigma, St. Louis). The antibiotic solution was mixed and delivered using a stainless tube without prior sedation of the mice. All the antibiotic cocktail used in this study was freshly prepared. The schedule of the experimental procedure and timeline is shown in Fig. 7.

### 16 S rRNA gene sequencing and data analysis

To determine whether antibiotic treatment altered the microbiome, gene-sequencing analysis of 16 S rRNA on we performed high-throughput fecal microbiotal DNA isolated from antibiotic-treated group and control group mice. The feces of mice were collected for 16 S detection on the last morning of weeks 1, 2, and 3, respectively, and the collection and detection methods were similar to those reported in our previous studies [25]. Briefly, genomic RNA was extracted from 200 mg of feces by the phenol-chloroform method and the mass and concentration were determined. The V3-V4 region of 16 S rRNA gene was amplified by PCR using Phusion Hot Start Flex 2×Master Mix primers (341 F 5′-CCTACGGG NGGCWGCAG-3′, 805R 5′- GACTACHVGGGTATCTAATCC-3’). The library construction results were quantitatively analyzed by Qubit and Agilent 2100 analyzers. An insert sequence (275 ~ 450 bp) was selected and sequenced by NovaSeq sequencing platform. Paired-end data overlap was performed using FLASH software (version 1.2.11). Clean data were obtained using Fqtrim (v0.94). Data was filtered using Vsearch (v2.3.4). Specific features and sequences were further obtained by split amplicon denoising algorithm (DADA2) and ASV analysis. The relative abundance of each feature was normalized using the SILVA (version 132) classifier. BLAST clustered features into operational taxonomic units (OTUs) with 97% similarity. Gene prediction and construction of non-redundant gene sets were performed using MetaGeneMark. The species annotation information of each gene were obtained by MyTaxa and related databases, and the species abundance table of different taxonomic levels obtained by combining with the gene abundance table. Bioinformatic analysis was performed using the OmicStudio tools at https://www.omicstudio.cn/tool.

### Histomorphology

Hematoxylin-eosin staining (H&E) was used to detect the intestinal morphology of mice. The fresh intestinal tissue of mice was fixed in 4% polyformaldehyde fixing solution for at least 24 h, and then the tissue was sliced to 4 μm thickness using a microtome after fixation, dehydration, paraffin infiltration, embedding and other steps. The sections were dried, and paraffin wax was removed before H&E staining was applied. Imaging was performed with NanoZoomer Digital Pathology (NDP), and quantitative analysis was performed using the software NDP View 2.

### Immunohistochemistry

Immunohistochemistry (IHC) was used to assess COX-2 and iNOS expression. After the paraffin sections were hydrated and dewaxed, the antigen was repaired using the thermal repair method. Then 3% hydrogen peroxide was added to the tissue surface to block the endogenous peroxidase activity, and 5% goat serum was added for serum blocking. The sections were incubated at room temperature and washed with PBS for three times. Then, The sections were incubated at 4 °C overnight with COX-2 or iNOS primary antibody. On the second day, after washing with PBS three times, The sections were incubated with HRP-conjugated secondary antibody at room temperature for 60 min. 3,3’-diaminobenzidine tetrachloride (DAB) was used for color development, and hematoxylin counterstaining was performed. The COX-2 (ET1610-23) and iNOS (ER1706-89) were obtained from HUABIO (Hangzhou, China). The HRP-conjugated Anti Rabbit/Mouse IgG was purchased from Beyotime (Shanghai, China). The images were collected using an optical microscope, and the positive area was quantitatively analyzed by ImageScope software.

### Real-time polymerase chain reaction

Real-time polymerase chain reaction (PCR) was used to detect the expression of cytokines. Total RNA was extracted from intestinal tissues using an RNA extraction kit, and the concentration of total RNA was determined by NanoDrop 2000 (Thermo Fisher Scientific, USA). cDNA was synthesized using a Synthesis Kit (Thermo Fisher Scientific, USA). The PCR conditions were as follows: template denaturation at 95 ℃ for 5 min and 45 amplification cycles of 95 ℃ for 10 s, 55 ℃ for 30 s, and 72 ℃ for 10 s. The gene expression levels were normalized to β-actin and analyzed according to the comparative Ct (2-ΔΔCt) method. Amplification specificity Primers were designed based on NCBI (http://www.ncbi.nlm.nih.gov) related mouse gene sequences in Genbank using Primer Premier 5 software. The primer sequences were as follows: IL-1β: forward 5′-TCATGGGATGATGATAACCTGCT-3′ and reverse 5′-CCCATACTTTAGGAAGACACGGATT-3′. IL-6: forward 5′-CTTTTGATATATGGAAT-3′ and reverse 5′-CCAGTTTGGTAGGCATCCATC-3′. TNF-α: forward 5′-CCCTCACACTCAGATCATCTTC-3′ and reverse 5′-GTTGGTTGTCTTTGAGATCCAT-3′.

### Mendelian randomization method

MR Studies used different gut microbiota as exposure factors and IL-6, IL-1β, TNF-α and COX-2 as outcome variables to explore the causal effect of gut microbiota on inflammatory response. As an instrumental variable, microbia-related SNPs should meet three conditions:

i) there is an association between instrumental variables and risk factors;
ii) There is no relationship between instrumental variable and confounders, and they are independent of each other;
iii) There is no direct relationship between instrumental variable and outcome variables.

The genetic instruments for inflammatory cytokines (IL-6, IL-1β, TNF-α and COX-2) were obtained from a large-scale genome-wide association study (GWAS) [26]. The GWAS study included up to 8,293 Finnish individuals from three independent population cohorts; The phenotypes of *Actinobacteria*, *Actinobacteria* and *Lactobacillus* are derived from a published study by Alexander Kurilshikov et al. [27], which included 18,340 individuals from 24 cohorts and multiple ethnicities. *Oxyphotobacteria*, *Comamonas* and *Serratia* did not find SNPs that could be used for MR Analysis. The aggregate data mentioned above are publicly available, and the relevant ethics have been approved by their respective agency review committees.

The screened IVs studied should be closely correlated with exposure (*P* < 5 × 10^− 8^). If two or more SNPs could not be obtained by this screening criterion, *P* < 5 × 10^− 6^ was used instead. Subsequently, the F-number of IVs was calculated to prevent weak tools (F < 10) from influencing the results (Supplementary Table S6). The value of F is calculated as F = R^2^ (n-k-1)/[k (1-R^2^)] [28]. LD clustering of identified SNPs was performed to remove linkage unbalance (R^2^ > 0.8 in 10,000 kb window). In addition, we use the PhenoScanner database (http://www.phenoscanner.medschl.cam.ac.uk/) to detect any genome-wide SNPs significantly associated with traits and deleted the SNPs that were directly related to the outcome variables. All palindromic SNPs are removed by ensuring that the SNPs reference the same allele. The IVs obtained from the final screening are shown in the supplementary materials (Supplementary Table S7 and S8).

MR Analysis is mainly carried out through the R package “TwoSampleMR”. The methods include IVW, MR-Egger regression, Weighted median approach, Simple mode, and Weighted mode methods. Among them, the MR Analysis was mainly carried out by IVW method, with *P* < 0.05 was regarded as statistically significant evidence [29]. This method assumes that horizontal pleiotropy does not exist in the study and is the most effective method at present. Cochran Q statistics are used to quantify heterogeneity; Q > 0.05 indicated no significant heterogeneity, and an IVW fixed-effect meta-analysis was used to combine Wald estimates for each SNP. When Q < 0.05, a random effects model is used [30].

MR-Egger regression assesses the horizontal pleiotropy of SNPs used as IVs by the distance between the intercept term and 0, *P* > 0.05 indicated that there was no horizontal pleiotropy in the identified IVs. The “MR-PRESSO outlier test” was used to remove the abnormal outlier SNPs with a distribution value of 10,000, and the stability of the corrected results was evaluated. “leave-one-out” sensitivity analysis can be used to assess the effect of individual genetic variants on the results of causal estimates, thereby assessing the robustness and reliability of the study. Finally, the results of MR Analysis were re-evaluated by removing SNPs that might be confounding factors.

### Statistical analysis

The results are expressed as means ± SD. Significant differences between groups were determined using one-way ANOVA tests. All statistical analyses were performed with GraphPad Prism 13.0 (GraphPad Software, San Diego, USA).

### Electronic supplementary material

Below is the link to the electronic supplementary material.

Supplementary Material 1

## Data Availability

All data generated or analyzed during this study were included in this article. The metagenome raw sequence dataset has been uploaded to the NCBI database under accession number PRJNA918963 (https://www.ncbi.nlm.nih.gov/bioproject/?term=PRJNA918963).

## Abbreviations

GF: Germ-free
MDR: Multidrug resistance
