# Supplementary material for: Effects of antibiotic cocktail on the fecal microbiota and their potential correlation of local immune response
Source: BMC Microbiol. 2024 Jul 31;24:283. doi: 10.1186/s12866-024-03424-z (PMC11290084; doi:10.1186/s12866-024-03424-z)
Supplement: Supplementary file 1 — Supplementary Material 1 [file 12866_2024_3424_MOESM1_ESM.docx]

***Supplementary file***

***
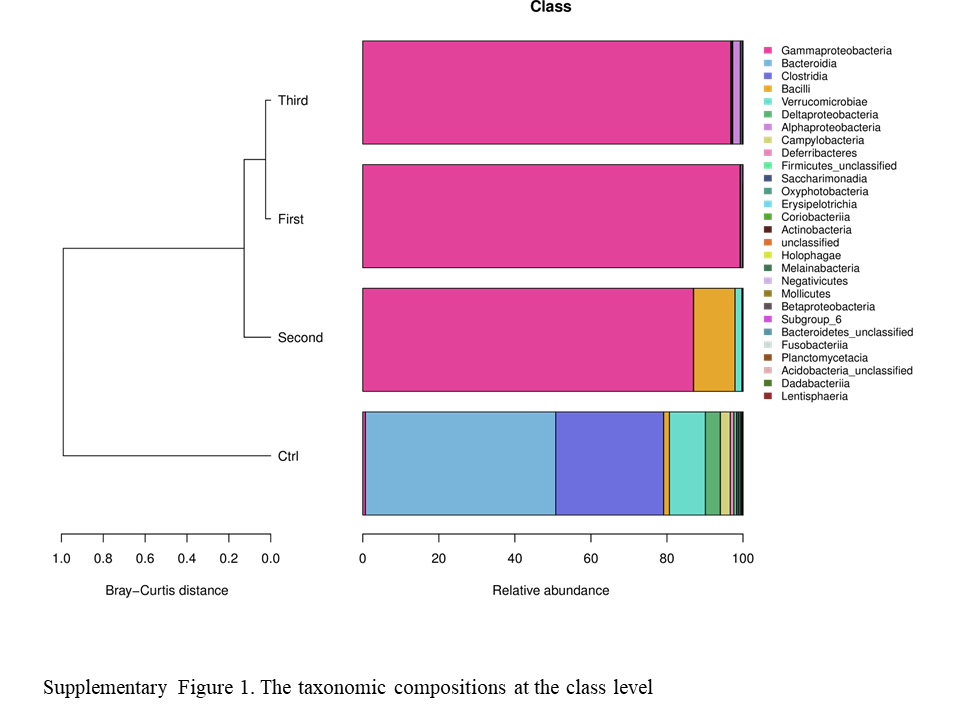
***

**Figure S1**. The taxonomic compositions at the class level

***
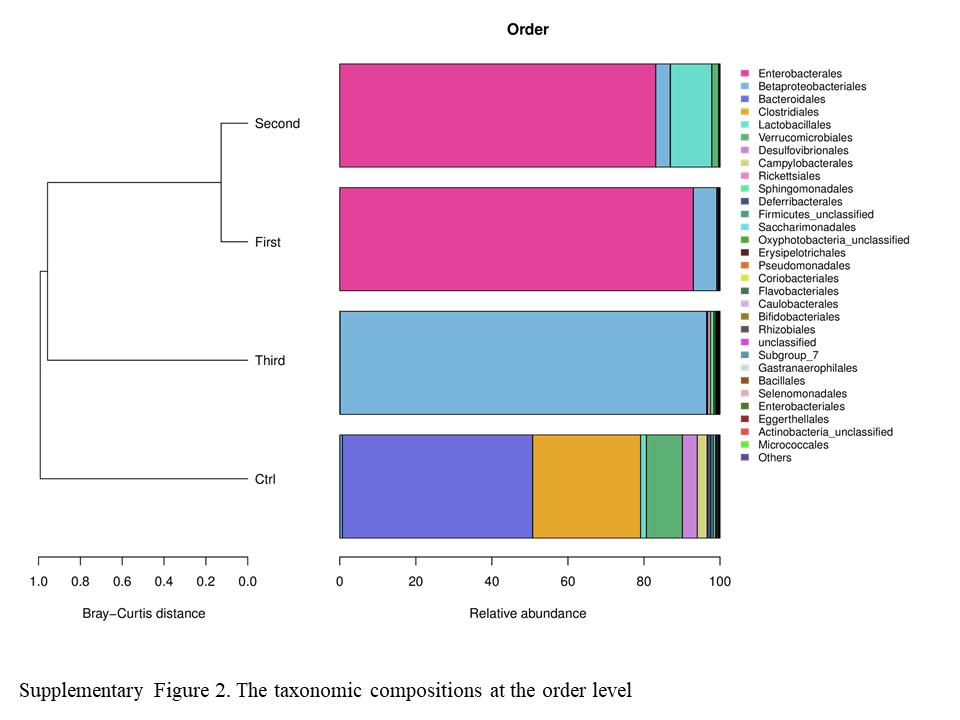
***

**Figure S2**. The taxonomic compositions at the order level

***
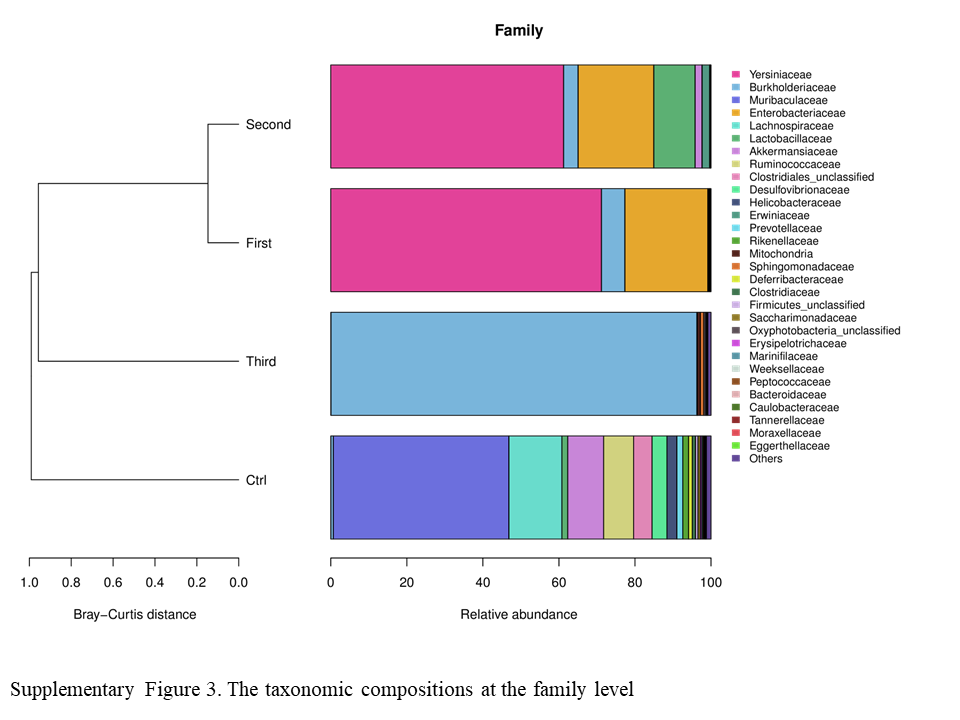
***

**Figure S3**. The taxonomic compositions at the family level


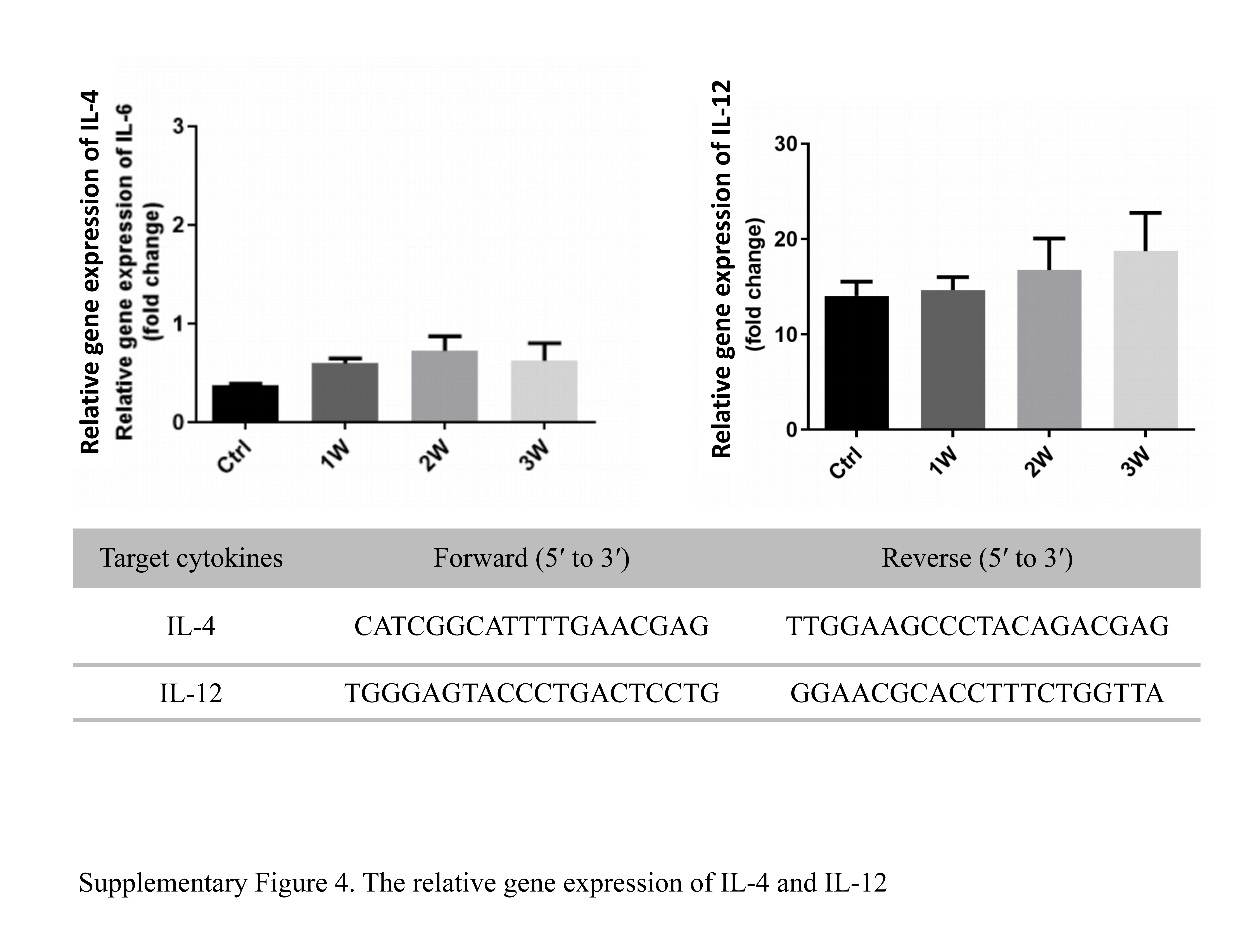


**Figure S4**. Relative gene expression of IL-4 and IL-12 in colon


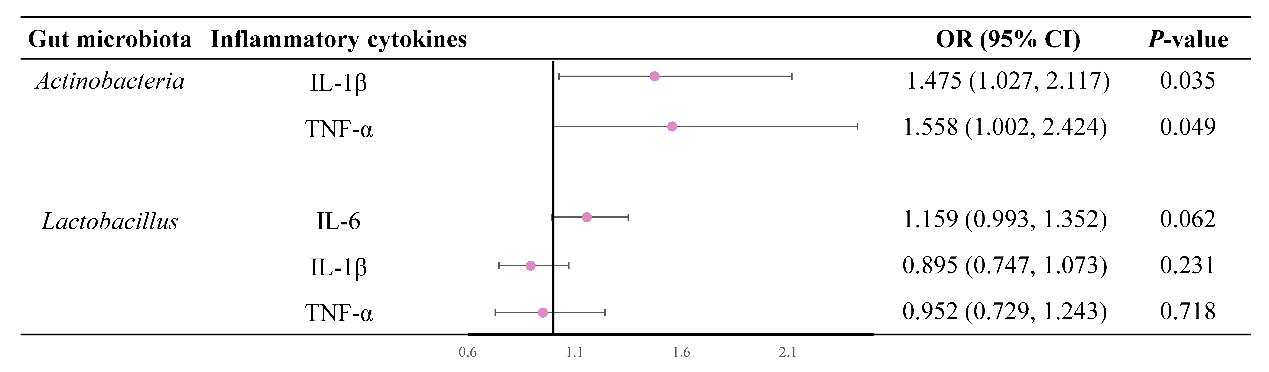


**Figure S5.** Leave-one-out analysis of associations of gut microbiota on inflammatory cytokines

*
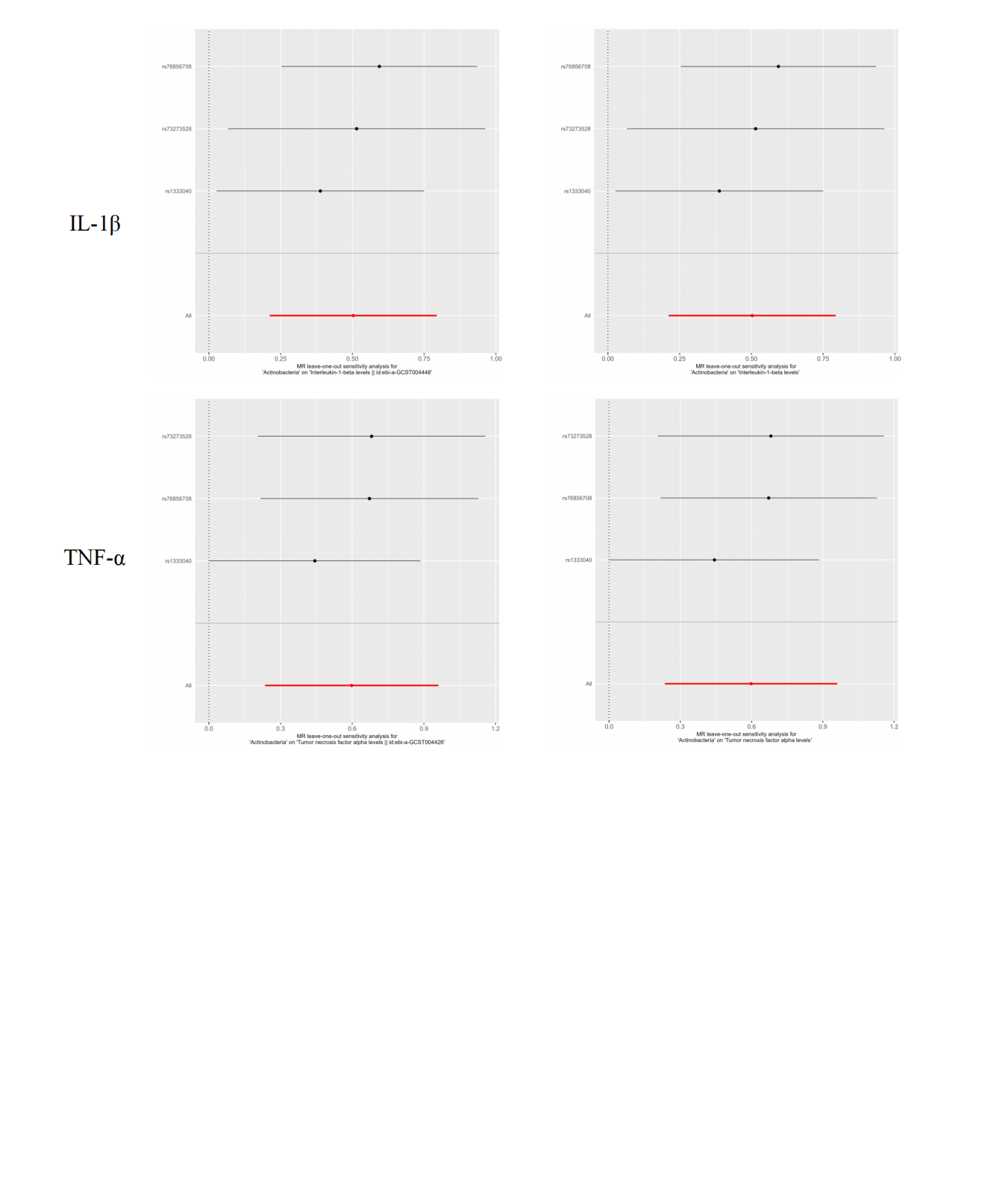
*

**Figure S6.** Leave-one-out analysis of associations of *Actinobacteria* on inflammatory cytokines

*
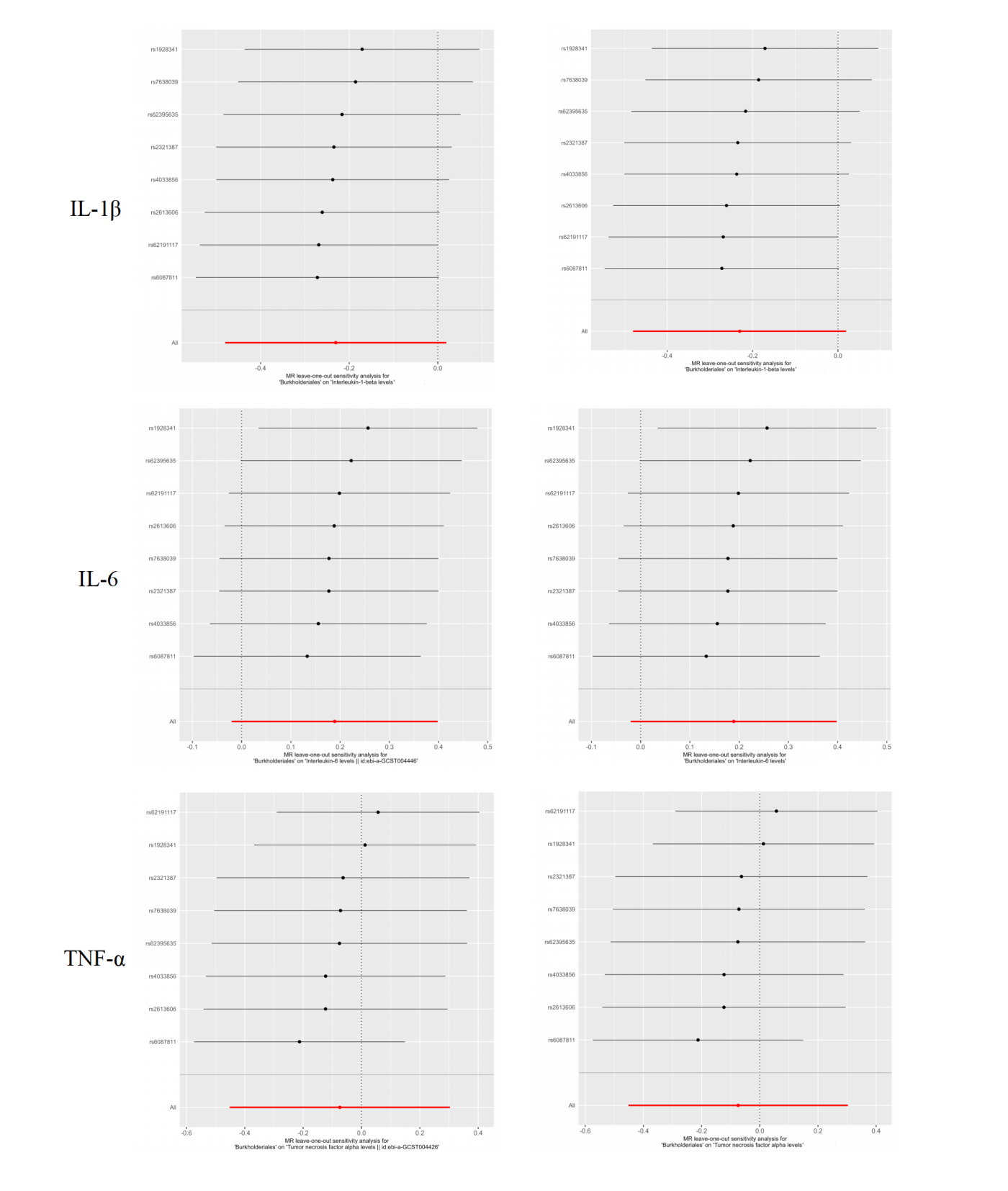
*

**Figure S7.** Leave-one-out analysis of associations of *Burkholderiales* on inflammatory cytokines

*
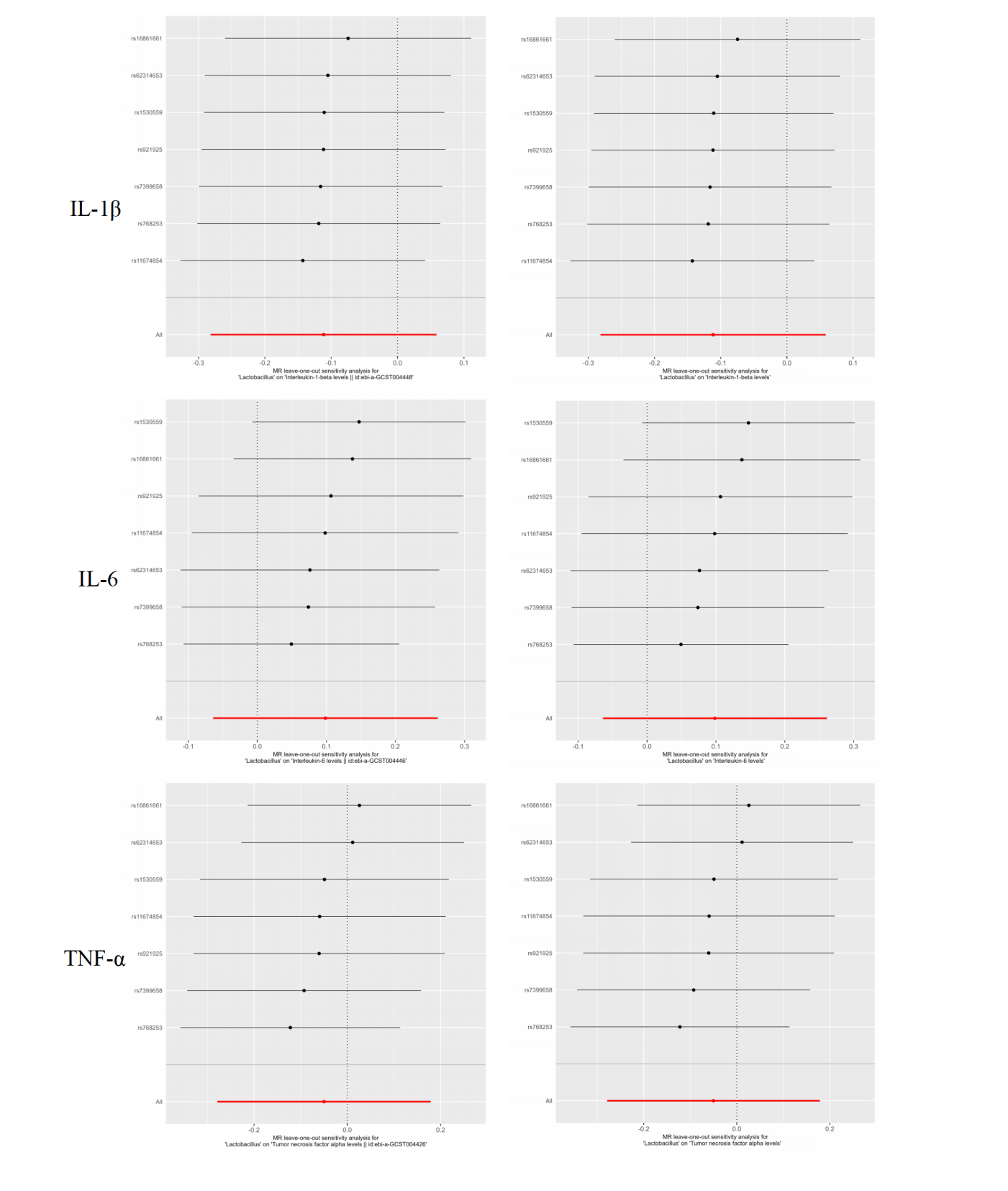
*

**Figure S8.** Leave-one-out analysis of associations of *Lactobacillus* on inflammatory cytokines

**Table S1.** Characteristics of the genetic variants that were used as the instrumental variables for *Actinobacteria**

| **SNP** | **Beta** | **SE** | **EAF** | **Effect allele** | **Other allele** | **P-value** |
| --- | --- | --- | --- | --- | --- | --- |
| rs10752777 | 0.110 | 0.024 | 0.116 | A | T | 3.25E-06 |
| rs75101555 | 0.367 | 0.078 | 0.982 | G | C | 2.60E-06 |
| rs1333040 | 0.074 | 0.016 | 0.426 | T | C | 3.17E-06 |
| rs76856708 | -0.329 | 0.070 | 0.038 | C | T | 2.61E-06 |
| rs72831623 | 0.197 | 0.037 | 0.053 | A | G | 1.08E-07 |
| rs73273528 | 0.267 | 0.055 | 0.966 | T | C | 9.58E-07 |
| rs1884910 | -0.079 | 0.017 | 0.637 | G | C | 3.26E-06 |

**Table S2.** Estimates of causal effect of gut microbiota on inflammatory cytokines.

| **Exposure** | **Outcome** | **Method** | **No. of SNPs** | **OR (95% CI)** | ***P*-value** |
| --- | --- | --- | --- | --- | --- |
| *Actinobacteria* | IL-1β | MR Egger | 3 | 1.310 (0.784, 2.188) | 0.490 |
|  |  | Weighted median |  | 1.621 (1.081, 2.431) | 0.020 |
|  |  | Simple mode |  | 1.624 (1.026, 2.570) | 0.174 |
|  |  | Weighted mode |  | 1.619 (0.985,2.662) | 0.198 |
| *Actinobacteria* | TNF-α | MR Egger | 3 | 1.328 (0.709, 2.489) | 0.538 |
|  |  | Weighted median |  | 1.570 (0.976, 2.526) | 0.063 |
|  |  | Simple mode |  | 1.558 (0.888, 2.734) | 0.262 |
|  |  | Weighted mode |  | 1.558 (0.882, 2.751) | 0.266 |
| *Burkholderiales* | IL-6 | MR Egger | 8 | 1.608 (0.759, 3.410) | 0.262 |
|  |  | Weighted median |  | 1.261 (0.969, 1.643) | 0.085 |
|  |  | Simple mode |  | 1.292 (0.828, 2.016) | 0.296 |
|  |  | Weighted mode |  | 1.288 (0.830, 1.998) | 0.296 |
| *Burkholderiales* | IL-1β | MR Egger | 8 | 1.022 (0.416, 2.511) | 0.963 |
|  |  | Weighted median |  | 0.843 (0.603, 1.180) | 0.321 |
|  |  | Simple mode |  | 0.912 (0.557, 1.493) | 0.724 |
|  |  | Weighted mode |  | 0.926 (0.568, 1.510) | 0.767 |
| *Burkholderiales* | TNF-α | MR Egger | 8 | 1.885 (0.494, 7.200) | 0.389 |
|  |  | Weighted median |  | 0.928 (0.602, 1.431) | 0.734 |
|  |  | Simple mode |  | 1.086 (0.507, 2.325) | 0.838 |
|  |  | Weighted mode |  | 1.093 (0.505, 2.364) | 0.827 |
| *Lactobacillus* | IL-6 | MR Egger | 7 | 1.037 (0.566, 1.901) | 0.910 |
|  |  | Weighted median |  | 1.104 (0.896, 1.361) | 0.351 |
|  |  | Simple mode |  | 1.178 (0.844, 1.645) | 0.372 |
|  |  | Weighted mode |  | 1.165 (0.841, 1.615) | 0.394 |
| *Lactobacillus* | IL-1β | MR Egger | 7 | 0.698 (0.393, 1.238) | 0.274 |
|  |  | Weighted median |  | 0.896 (0.719, 1.115) | 0.323 |
|  |  | Simple mode |  | 0.900 (0.661, 1.255) | 0.528 |
|  |  | Weighted mode |  | 0.900 (0.654, 1.239) | 0.541 |
| *Lactobacillus* | TNF-α | MR Egger | 7 | 0.448 (0.214, 0.936) | 0.086 |
|  |  | Weighted median |  | 1.003 (0.743, 1.354) | 0.983 |
|  |  | Simple mode |  | 1.018 (0.616, 1.685) | 0.946 |
|  |  | Weighted mode |  | 1.018 (0.627, 1.654) | 0.944 |

**Table S3.** Heterogeneity analysis between gut microbiota and inflammatory cytokines through inverse variance weighted.

| **Exposure** | **Outcome** | ***P*-value** |
| --- | --- | --- |
| *Actinobacteria* | IL-1β | 0.478 |
| *Actinobacteria* | TNF-α | 0.486 |
| *Burkholderiales* | IL-6 | 0.607 |
| *Burkholderiales* | IL-1β | 0.805 |
| *Burkholderiales* | TNF-α | 0.215 |
| *Lactobacillus* | IL-6 | 0.271 |
| *Lactobacillus* | IL-1β | 0.958 |
| *Lactobacillus* | TNF-α | 0.366 |

**Table S4.** Horizontal pleiotropy analysis between gut microbiota and inflammatory cytokines.

| **Exposure** | **Outcome** | ***P*-value** |
| --- | --- | --- |
| *Actinobacteria* | IL-1β | 0.475 |
| *Actinobacteria* | TNF-α | 0.442 |
| *Burkholderiales* | IL-6 | 0.467 |
| *Burkholderiales* | IL-1β | 0.587 |
| *Burkholderiales* | TNF-α | 0.322 |
| *Lactobacillus* | IL-6 | 0.842 |
| *Lactobacillus* | IL-1β | 0.415 |
| *Lactobacillus* | TNF-α | 0.090 |

**Table S5.** F statistic between genetic instruments and exposure.

| **Exposure** | **Outcome** | **F-statistic (median)** | **F-statistic**  **(minimum)** | **F-statistic**  **(maximum)** |
| --- | --- | --- | --- | --- |
| *Actinobacteria* | IL-1β | 61.414 | 35.535 | 113.450 |
| *Actinobacteria* | TNF-α | 61.414 | 35.535 | 113.450 |
| *Burkholderiales* | IL-6 | 20.117 | 17.061 | 22.713 |
| *Burkholderiales* | IL-1β | 20.117 | 17.061 | 22.713 |
| *Burkholderiales* | TNF-α | 20.117 | 17.061 | 22.713 |
| *Lactobacillus* | IL-6 | 47.143 | 42.841 | 59.385 |
| *Lactobacillus* | IL-1β | 47.143 | 42.841 | 59.385 |
| *Lactobacillus* | TNF-α | 47.143 | 42.841 | 59.385 |

**Table S6.** Potential confounding variables in SNPs associated with gut microbiota and inflammatory cytokines.

| **Exposure** | **Outcome** | **SNP** | **Potential confounding trait** | ***P*-value** |
| --- | --- | --- | --- | --- |
| *Actinobacteria* | IL-1β | rs1333040 | Coronary artery disease | 0.00e+00 |
| *Actinobacteria* | TNF-α | rs1333040 | Coronary artery disease | 0.00e+00 |
| *Lactobacillus* | IL-6 | rs1530559 | Parkinsons disease | 1.70E-08 |
| *Lactobacillus* | IL-1β | rs1530559 | Parkinsons disease | 1.70E-08 |
| *Lactobacillus* | TNF-α | rs1530559 | Parkinsons disease | 1.70E-08 |

**Table S7.** Characteristics of the genetic variants that were used as the instrumental variables for *Burkholderiales**

| **SNP** | **Beta** | **SE** | **EAF** | **Effect allele** | **Other allele** | ***P*-value** |
| --- | --- | --- | --- | --- | --- | --- |
| rs1928341 | -0.051 | 0.011 | 0.422 | G | A | 4.52E-06 |
| rs62191117 | 0.068 | 0.013 | 0.206 | A | G | 2.79E-07 |
| rs7638039 | 0.058 | 0.013 | 0.767 | T | C | 4.84E-06 |
| rs2367850 | 0.063 | 0.013 | 0.779 | G | C | 1.24E-06 |
| rs4033856 | -0.083 | 0.017 | 0.871 | T | C | 5.67E-07 |
| rs62395635 | 0.110 | 0.024 | 0.929 | T | C | 2.90E-06 |
| rs2613606 | 0.050 | 0.011 | 0.394 | T | C | 4.13E-06 |
| rs72747231 | -0.134 | 0.029 | 0.042 | C | G | 3.76E-06 |
| rs2321387 | -0.051 | 0.0110 | 0.470 | G | A | 3.26E-06 |
| rs6087811 | -0.102 | 0.020 | 0.919 | T | G | 2.88E-07 |

**Table S8.** Characteristics of the genetic variants that were used as the instrumental variables for *Lactobacillus**

| **SNP** | **Beta** | **SE** | **EAF** | **Effect allele** | **Other allele** | ***P*-value** |
| --- | --- | --- | --- | --- | --- | --- |
| rs16861661 | -0.183 | 0.038148 | 0.950 | G | A | 1.28E-06 |
| rs11674854 | -0.085 | 0.01765 | 0.427 | C | T | 1.59E-06 |
| rs1530559 | 0.080 | 0.017821 | 0.432 | G | A | 4.93E-06 |
| rs62314653 | 0.188 | 0.039459 | 0.937 | C | A | 2.24E-06 |
| rs768253 | -0.079 | 0.017179 | 0.391 | T | G | 4.25E-06 |
| rs7399658 | -0.107 | 0.022188 | 0.216 | G | A | 3.12E-06 |
| rs328312 | 0.082 | 0.016944 | 0.553 | T | A | 1.41E-06 |
| rs921925 | 0.099 | 0.020323 | 0.738 | A | C | 9.72E-07 |
| rs6092149 | -0.080 | 0.017149 | 0.443 | A | T | 3.29E-06 |
